# Development of a Double Nanobody-Based Sandwich Immunoassay for the Detecting Staphylococcal Enterotoxin C in Dairy Products

**DOI:** 10.3390/foods10102426

**Published:** 2021-10-13

**Authors:** Yanwei Ji, Lili Chen, Yingying Wang, Kaihui Zhang, Haofen Wu, Yuan Liu, Yanru Wang, Jianlong Wang

**Affiliations:** College of Food Science and Engineering, Northwest A&F University, Xianyang 712100, China; jiyanwei@nwsuaf.edu.cn (Y.J.); nancychen2000@126.com (L.C.); wangyingying1123@163.com (Y.W.); zhangkaihui@nwafu.cn.com (K.Z.); whf@nwafu.edu.cn (H.W.); liuyuan01@nwafu.edu.cn (Y.L.); yanruwang22@163.com (Y.W.)

**Keywords:** Staphylococcal enterotoxin C, nanobody, sandwich ELISA, phage display, dairy products

## Abstract

Staphylococcal enterotoxins (SEs) represent the leading reason for staphylococcal food poisoning (SFP) and various other diseases. Reports often indicate Staphylococcal enterotoxin C (SEC) as the most frequently found enterotoxin in dairy products. To minimize consumer exposure to SEC, this paper aimed to create a sandwich enzyme-linked immunosorbent assay (ELISA) based on nanobodies (sandwich Nbs-ELISA) to accurately detect SEC in dairy products without the influence of staphylococcal protein A (SpA). Therefore, after inoculating a Bactrian camel with SEC, a phage display Nb library was created. Eleven Nbs against SEC were identified in three biopanning steps. Based on their affinity and pairing level, a sandwich Nbs-ELISA was developed using the C6 anti-SEC Nb as the capture antibody, while the detection antibody was represented by the C11 phage display anti-SEC Nb. In optimal conditions, the quantitative range of the present sandwich ELISA was 4-250 ng/mL with a detection limit (LOD) of 2.47 ng/mL, obtained according to the blank value plus three standard deviations. The developed technique was subjected to specific measurements, revealing minimal cross-reactivity with *Staphylococcus aureus* (*S. aureus*), Staphylococcal enterotoxin A (SEA), Staphylococcal enterotoxin B (SEB), and SpA. The proposed method exhibited high specificity and an excellent recovery rate of 84.52~108.06% in dairy products. Therefore, the sandwich Nbs-ELISA showed significant potential for developing a specific, sensitive technique for SEC detection in dairy products.

## 1. Introduction

*Staphylococcus aureus* (*S. aureus*) is a foodborne pathogen abundant in nature and can cause severe staphylococcal food poisoning (SFP). The pathogenicity of *S. aureus* depends on the production of low molecular weight alkaline globular protein exotoxins, namely staphylococcal enterotoxins (SEs). These are small, water-soluble proteins that are highly stable, significantly temperature resistant, and display super antigenic activity [1]. Therefore, SEs remain active and pathogenic after treatment with general methods, with only a few micrograms necessary to cause SFP. In Europe, about 15–20% of food poisoning outbreaks (FPO) are caused by bacterial toxins, of which about 50% are related to the SEs produced by *S. aureus* [2]. The main SE serotypes include SEA, SEB, SECs, SED, and SEE. Of these, SECs include three subtypes—namely, C1, C2, and C3—and are most frequently found in dairy products [3,4,5]. Therefore, reliable, exceptionally sensitive methods are crucial for detecting SECs in dairy products, monitoring dairy safety, and aiding traceback investigations during SFP outbreaks.

Currently, the SE detection strategies mainly include molecular biological methods [6], immunological methods [7], and mass spectrometry [8]. Of these, immunological methods employing a combined reaction involving antigens and antibodies, are commonly used for quantitatively identifying SEs in complex food matrixes [9]. The traditionally used enzyme-linked immunosorbent assay (ELISA) presents advantages, such as simple operation, low cost, and mature technology [10]. Currently, polyclonal or monoclonal antibodies are used as recognition elements for most ELISA methods. However, for conventional monoclonal antibodies, the preparation is time-consuming and labor-intensive, yielding a low output. Moreover, since staphylococcal protein A (SpA) presented or secreted on the surface of *S. aureus* may bind to the fragment crystallizable region (Fc) of the monoclonal antibody with high affinity, some challenges may arise, such as false-positive results, impeding ELISA application [11,12].

A nanobody (Nb), a variable heavy-chain domain (VHH), is a single-chain antibody with natural light-chain deletion found in Camelidae (camels, alpacas, and llamas) and sharks [13,14]. It presents an oval crystal structure with a 2.5 nm diameter and a 4 nm length, all of which are at the nanometer level. The molecular weight of an Nb is about 15 kDa, and its volume is only one-tenth that of traditional monoclonal antibodies [15,16]. It is the smallest antibody fragment discovered so far. Nbs presents the following advantages: (1) Good water solubility allows for easy, large-scale VHH expression in a variety of systems at a low production cost. Moreover, Nbs are beneficial for maintaining functionality and improving recovery rates. (2) The presence of disulfide bonds in the Nbs renders them highly stable in high-pressure, high-temperature, denaturing, and other conditions, which is conducive to antibody preservation. (3) Strong affinity. The complementarity-determining region 3 (CDR3) is a vital antigen-binding site for antibodies. The Nb CDR3 is long and flexible and can form an exposed convex ring structure [17,18,19]. Moreover, due to their small size, Nbs can reach the gaps and cracks on the surface of the antigen, which is not possible for traditional monoclonal antibodies, allowing for better amalgamation with the antigen. Compared with monoclonal antibodies, Nbs can effectively avoid false-positive results caused by SpA binding due to the natural lack of Fc terminal recognition sites [12]. Nbs have attracted increasing research attention due to their many excellent characteristics and functions and have been applied in the food science field, especially for food safety detection [20,21,22].

**Scheme 1:**
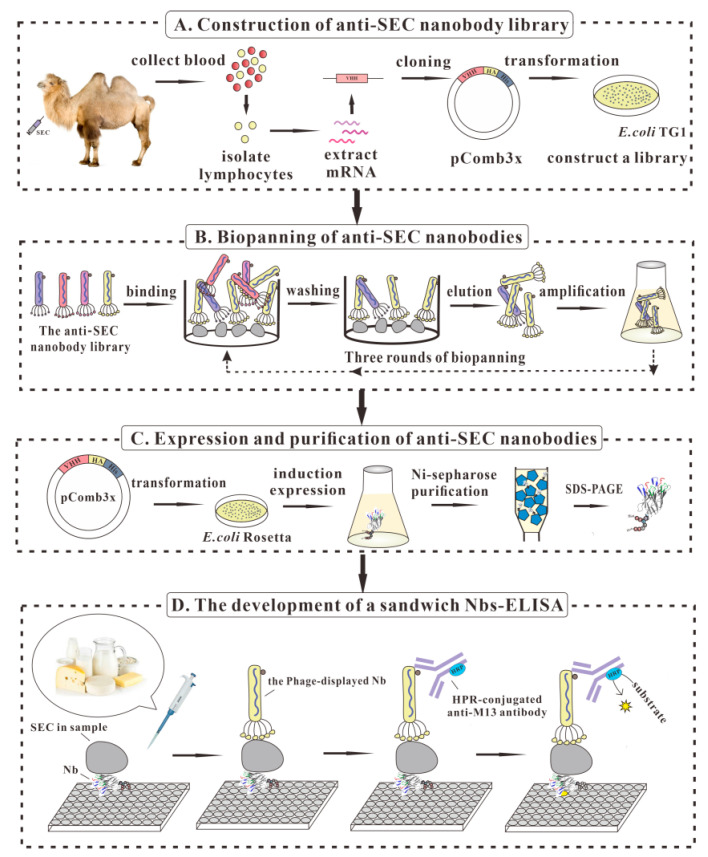
Schematic diagram of the sandwich Nbs-ELISA. (**A**), construction of anti-SEC nanobody library. (**B**), Biopanning of anti-SEC nanobodies. (**C**), Expression and purfication of anti-SEC nanobodies. (**D**), The development of a sandwich Nbs-ELISA.

This work aims to construct a sandwich Nbs-ELISA to sensitively and specifically detect SECs without SpA in dairy products. The principle of the sandwich Nbs-ELISA is presented in Scheme 1. This study constructed an anti-SEC Nb library by inoculating a Bactrian camel with SEC as the immunogen, after which 11 anti-SEC Nbs were panned from the phage display, Nb library using a biopanning approach. The method uses phage display Nbs as reporter elements, improving the sensitivity since each phage contain about 2,700 copies of the pVIII capsid protein for signal amplification, as well as four to five protein (pIII, protein VIII) copies on either end of every particle to improve affinity [23,24]. The method uses Nbs as recognition elements, avoiding false-positive results caused by the combination with SpA in the traditional ELISA method using monoclonal antibodies as recognition elements. The new technique shows significant application promise in dairy and agricultural products.

## 2. Materials and Methods

### 2.1. Materials

The Academy of Military Medical Sciences (Beijing, China) provided three serological types of SEs (SEC, SEB, and SEA) derived from *S. aureus*. The SuperScript III, First-Strand Synthesis SuperMix RT-PCR kit and the TRIzol reagent were obtained from Thermo Fisher Scientific (Waltham, MA, USA), Invitrogen (USA), the Shanghai Jingkang Biotechnology Co. (Shanghai, China), and Costar (Cambridge, MA, USA). The pComb3x vector was supplied by MRC (Cambridge, England). The horseradish peroxidase (HRP)-labeled anti-M13 antibodies, M13K07 helper phage, *Escherichia coli* (*E. coli*) TG1, HA-tag antibody-HRP, and Ni-affinity chromatography were acquired from Thermo Fisher Scientific (Carlsbad, CA, USA). Sigma-Aldrich (St. Louis, MO, USA) provided the ovalbumin (OVA), bovine serum albumin (BSA, ~66 kDa), Freund’s complete adjuvant (cFA) and incomplete adjuvant (iFA), as well as 3,3′,5,5′-Tetramethylbenzidine (TMB), isopropyl-β-D-thiogalactopyranoside (IPTG), and polyethylene glycol (PEG-8000). The Taq DNA polymerase, T4 DNA ligase, *Sfi* I Fast Digest restriction enzymes, and the synthesis kit for first-strand cDNA were provided by Takara Co. (Dalian, China). All other reagents were used as received and of analytical grade. For the duration of the study, a Milli-Q system was used to produce ultrapure water.

### 2.2. Bactrian Camel Immunization

An emulsified SEC standard (100 μg) mixture and the same quantity of cFA was injected into a healthy male Bactrian camel as the main immunization (day 1). In subsequent immunizations (4 times, at 14 day, 28 day, 42 day, and 56 day), the emulsified SEC standard mixture (100 µg) and an equal volume of iFA were subcutaneously injected into the camel. Furthermore, in total, 200 mL peripheral blood was collected weekly after (70 day) the final immunization. The lymphocytes were isolated using Ficoll PLUS and stored at −80 °C for future use. To evaluate the immune response, serum from the immunized camel was separated to monitor the anti-SEC serum IgG antibody titer via indirect ELISA.

### 2.3. Constructing the Phage Display Nb Library

A method outlined in a previous study [25,26] was used to construct the Nb phage display library, the principle of which is shown in Scheme 1A. TRIzol Reagent was employed to obtain the total RNA from the 10^8^ peripheral blood lymphocytes. Next, a reverse transcription kit was used to synthesize first-strand complementary DNA (cDNA) from the total mRNA. Two-step nested PCR was utilized for VHH gene amplification using cDNA as a template, while the antibody VH genes were amplified using a pair of primers (CALL001 and CALL002). Furthermore, the 700 bp PCR products were subjected to agarose gel purification. This process was followed by repeating the PCR analysis for VHH gene amplification using the 700 bp fragments as a template, along with nested VHH-Back primers (5′-CAT GCC ATG ACT CGC GGC CGG CCT GGC CGG AGA CGG TGA CCW GGG T-3′) and VHH-For primers (5′-CAT GCC ATG ACT GTG GCC CAG GCG GCC GAG TCT GGR GGA GG-3′), which included the *Sfi* I double-digested restriction sites. Next, an agarose gel purification kit was used to purify the subsequent *Sfi* I double-digested PCR products (400 bp). *Sfi* I enzyme digestion, the pComb3x vector, and PCR products were ligated and continuously electrotransformed into *E. coli* TG1-competent cells. They were placed on a yeast extract growth medium (YT), 2× tryptone agar plate with 2% (*w/v*) glucose, as well as 100 μg/mL ampicillin. Next, the transformation efficiency was calculated via the plating and gradient dilution method. Next, the transformants were collected from the cultivation plates and recovered using the M13K07 helper phage to obtain a phage display Nb library, after which its size was estimated via LB-ampicillin plating onto agar plates, from which a random selection of 22 individual clones were used to assess the appropriate library insertion rate via PCR. Recombinant *E. coli* TG1 that included the pCombxss VHH plasmid was introduced into a 2 × YT mixture consisting of 100 μg/mL ampicillin, as well as 2% (*v/v*) glucose until the OD_600nm_ ≈ 0.6. Then, recombinant *E. coli* TG1 cells were combined with the M13K07 helper phage to recover and enrich the unique phage particles for 1 h at 37 °C. Next, 3000× *g* centrifugation was employed for 10 min to collect the recombinant *E. coli* TG1 cells, after which they were resuspended and cultivated overnight using a 2 × YT mixture containing 50 μg/mL kanamycin, as well as 100 μg/mL ampicillin. The cell culture was collected via a 15 min, 5000× *g* centrifugation process at 4 °C, after which a PEG/NaCl solution was mixed with the supernatant. After a 2 h incubation period on ice, the precipitation of the phages was accomplished via a 20 min, 12,000× *g* centrifugation process. Finally, 1 mL of phosphate-buffered saline (PBS) was used for phage particle resuspension, followed by quantification via phage titration. The remainder of the phages were kept at −80 °C for subsequent biopanning experiments.

### 2.4. Biopanning of the Phage Display Anti-SEC Nbs

The phage display Nbs against SEC were cycled through three consecutive rounds of binding selection with SEC coated onto 96-well microtiter plates as the capture antigen (Scheme 1B). For the first biopanning process, 100 μL SEC (1000 ng/mL) was used to coat sterile microplate wells at 4 °C overnight. Then, 3% BSA-PBS (350 μL/well) was added to the wells as a blocking buffer and subjected to incubation for 2 h at 37 °C After adding 100 μL of the Nb phage display library (1.0 × 10^11^ pfu), the mixture was incubated for 1 h at 37 °C while subjected to gentle shaking. Next, the unbound phages were removed with sterile PBST (a PBS and 0.05% Tween-20 mixture), and the process was repeated 15 times. Moreover, 0.2 M sterile glycine-HCl (at pH 2.2 and 100 μL/well) was used to elute the specifically bound phage particles for 8 min at 37 °C, followed by instant neutralization using Tris-HCl (15 μL/well, pH 9.5). The eluted phages were titered and amplified by infecting the *E. coli* TG1 for another biopanning sequence and subsequent panning. During the three panning rounds, the wells were coated overnight with 100 μL SEC in decreasing concentrations (1000 ng/mL, 500 ng/mL, and 250 ng/mL), while the phage input quantities remained constant (1.0 × 1011 pfu). Furthermore, two kinds of blocking buffers (3% BSA and 3% OVA in PBS buffer) were used alternately during the three rounds to decrease the nonspecific binding, after which a total of 96 independent clones were chosen at random from the agar plate from the third elution process. These clones were further amplified in 2 mL of 2 × YT medium for phage-ELISA with a coating of SEC (500 ng/mL). Compared to the uncoated wells, the colonies displaying at least a two-fold higher signal in the wells with SEC were considered positive. Then, the positive clones were sequenced with the gback sequencing primer.

### 2.5. Preparation of the Phage Display Nbs

The recombinant *E. coli* TG1 plasmid was inoculated into a liquid 2 × YT-Amp medium and incubated until reaching the logarithmic growth phase (OD_600_ = 0.6). This process was followed by a 15 min incubation period at 37 °C without shaking to facilitate M13K07 helper phage infection. The culture was subjected to another 37 °C incubation process for 45 min at 220 rpm in a shaker. The bacteria collected via a 10 min, 3000× *g* centrifugation process were immersed in a 2 × YT mixture composed of 100 μg/mL ampicillin, as well as 50 μg/mL kanamycin. The phage display Nbs formed overnight at 30 °C while subjected to shaking (220 rpm). The culture was exposed to a 15min, 10,000× *g* centrifugation process, during which the phage supernatant was amplified, followed by precipitation with a 1/5 quantity of 20% (*w/v*) PEG/NaCl. Next, the phages were subjected to a 20 min, 10,000× *g* centrifugation process, after which they were collected and suspended in PBS. The colony-forming units (CFU) were counted using titration.

### 2.6. The Purification and Expression of the Anti-SEC Nbs

After the recombinant plasmids of 11 different anti-SEC Nbs were converted to *E. coli* Rosetta cells, the individual colonies were collected to express the Nb (Scheme 1C). The cells were subjected to 37 °C incubation at 220 rpm in LB-Amp medium with 0.1% glucose ampicillin (100 μg/mL) until reaching an OD600 value of 0.6~0.8. Then, Nb expression was induced at 30 °C and 220 rpm for 8 h using 1 mM IPTG. The cells were centrifuged to obtain pellets and lysed with B-PER reagent, after which the solution was subjected to a 10 min, 8000× *g* centrifugation process. The obtained supernatant was subjected to 0.22 μm filtration and placed on a Ni-NTA Superflow chromatography column to purify the expressed Nbs. The anti-SEC Nbs were eluted with 500 mM imidazole solution, after which they were dialyzed and analyzed using SDS-PAGE.

### 2.7. Pairwise Selection for Sandwich Nbs-ELISA

The selection of matching detection and capture antibodies is essential to enhance the sensitivity of the sandwich ELISA. Furthermore, to determine the optimum paring of the Nbs for the sandwich ELISA, 11 anti-SEC Nbs were paired as capture antibodies with the corresponding 11 phage display Nbs. An SEC standard consisting of 0.01 M (100 μL/well) and 500 ng/mL PBS was added as negative and positive control solutions, respectively. The same method was used for the sandwich ELISA procedure. The optimal pair was considered as consisting of the most significant OD_450_ ratio obtained for the positive and negative control samples (P/N). The selected Nb pair was used for sandwich ELISA development.

### 2.8. Developing a Sandwich Nbs-ELISA for SEC Detection

The C6 anti-SEC Nb and C11 phage display anti-SEC Nb concentrations were optimized according to the pairwise selection findings, using a checkerboard method. The plates received separate coatings of the C6 capture Nbs diluted to 1 μg/mL, 2 μg/mL, 4 μg/mL, 8 μg/mL, and 16 μg/mL. Next, 100 μL of PBS buffer and 100 μL of SEC (500 ng/mL) were added as negative and positive control solutions, respectively, after blocking. Then, the wells were washed, and 100 μL of the C11 phage display Nb (1 × 106, pfu/mL 5 × 10^6^ pfu/mL, 2.5 × 10^7^ pfu/mL, 1.25 × 10^8^ pfu/mL, and 6.25 × 10^8^ pfu/mL) was added. Here, 100 μL of anti-SEC Nbs in PBS buffer was used to coat 96-well plates at 4 °C overnight for the sandwich ELISA. The anti-SEC Nbs solution was decanted, followed by the addition of 350 μL blocking buffer (5% nonfat milk in PBS) to each well for 2 h at 37 °C. A rinsing process using PBST was repeated three times, followed by a 45 min, 37 °C addition of 100 μL of the SEC serial dilutions to the wells. PBST was used to wash the wells three times, followed by a 45 min addition of 100 μL of the selected phage display Nbs at 37 °C. Next, 100 μL of the anti-M13 antibodies conjugated with HRP were added at 37 °C for 45 min. Finally, 100 μL of fresh TMB substrate was pipetted into the wells and subjected to a 15 min incubation period, after which 2 M H_2_SO_4_ solution was used to terminate the reaction. A microplate reader was then employed to read the microplate wells at 450 nm (Scheme 1D).

### 2.9. Cross-Reactivity Examination

The technique specificity was ascertained by spiking the sample extraction with two *S. aureus* strains, including *S. aureus* ATCC25923, *S.aureus* ATCC29213, as well as two additional types of SEs, namely SEA and SEB, and SpA.

### 2.10. Analysis of the Spiked Dairy Product

A series of SEC concentrations were spiked into dairy products (pure fresh milk, cheese, yogurt, and milk powder) using standard addition methods to validate the performance of the existing techniques. The samples were sourced from supermarkets and dairy ranches in Yangling, China. Commercial sandwich ELISA kits were used to verify that the samples were free of SEC, while the assessment only utilized negative samples. Next, 5 g solid dairy samples (cheese and milk powder) and 5 mL liquid milk samples were rubbed and dispersed in 45 mL PBS buffer. The samples were subjected to a 10 min, 5000× *g* centrifugation process at 4 °C and transferred to a new, 1.5 mL tube after removing the upper layer of fat. Then, the ELISA assay created in this study was used to evaluate 100 μL samples spiked with SEC, while the negative control was represented by an unspiked sample.

## 3. Results

### 3.1. Anti-SEC Nb Library Construction

**Figure 1:**
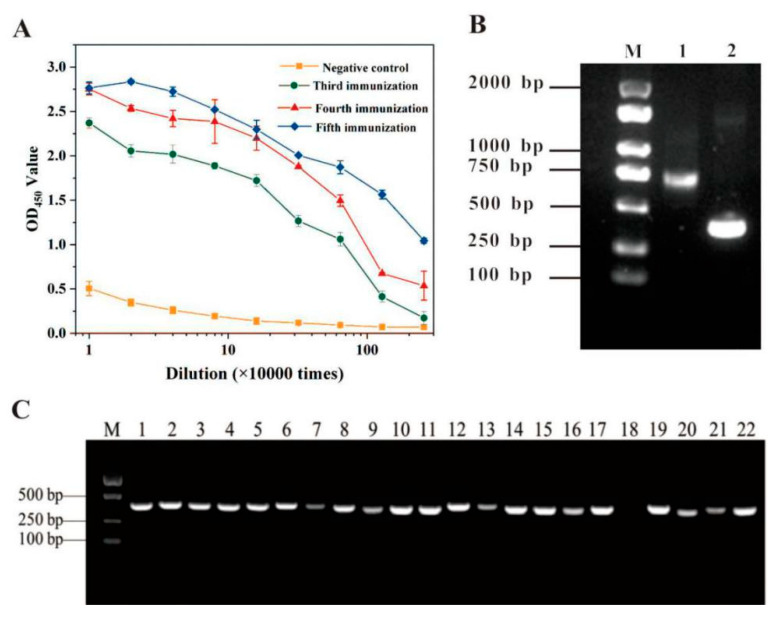
SEC-specific Nb library construction. (**A**) Anti-SEC serum via indirect ELISA. (**B**) The VHH genes were acquired via two-step PCR and run on an agarose gel. The initial fragments of the PCR display a band at approximately 750 bp. Secondary PCR was used to amplify the Nb genes, showing a 400 bp band. (**C**) The randomly selected 22 individual clones for estimating the appropriate insertion rate using colony PCR.

Highly pure SEC was injected five times into a healthy, male Bactrian camel to generate SEC-specific, high affinity, binding Nbs. Antibody affinity should be improved due to somatic hypermutation and antigen-driven clonal selection during the immune process [27,28]. To evaluate the immune response against SEC, the titration of the camel serum was achieved via indirect ELISA (with unimmunized serum as a negative control). According to Figure 1A, the anti-SEC titers showed an increase in reactivity toward SEC in conjunction with the immunization time, reaching an anti-SEC titer of 1:2,560,000 (OD450 > 1) after the fifth round of inoculation, suggesting that the camel produced a good immune response to the SEC. After acquiring the total RNA of the lymphocyte cells, it was transformed into cDNA using reverse transcription. Nb gene amplification was achieved via the two-step nested PCR approach, with cDNA synthesized as the template, while the CALL001 and CALL002 primer pair in the leading region was used to amplify the initial PCR products. The results are shown in Figure 1B, indicating that a DNA target band of approximately 700 bp was obtained after the first round of PCR, and about 450 bp fragments were obtained after the second round using the initial PCR products as a template (Figure 1B). Next, the VHH fragments were digested with the *Sfi* I restriction enzyme, ligated into pComb3x vector plasmids and transferred to *E. coli* TG1-competent cells. After the insertion rate and size of the library were estimated, the construction of the library was completed. As shown in Figure 1C, a random selection of 22 clones was analyzed using colony PCR to determine the appropriate insertion rate of the library, which was determined as 95.5%, indicating successful VHH library construction. A colony count showed that the library size was calculated as 5.2 × 10^8^ CFU. The overall size of the library size is essential in high-affinity clone acquisition. Therefore, this study successfully constructed a high-quality, immunized phage display Nb library for subsequent Nbs biopanning against SEC.

### 3.2. Biopanning of the Anti-SEC Nbs

**Figure 2:**
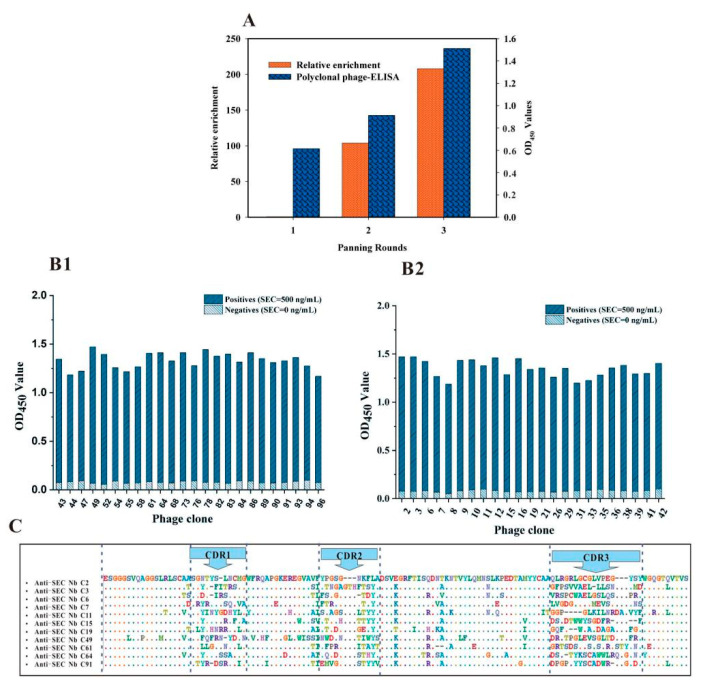
Biopanning of the anti-SEC Nbs. (**A**) The enrichment of the eluted phage during the biopanning cycles. (**B1**,**B2**) Screening and identification of the anti-SEC Nbs via indirect phage-ELISA. (**C**) The anti-SEC Nbs amino acid sequence alignment was categorized into 11 families due to significantly diverse amino acid sequences in the CDR3.

The phage display Nb library was established after recovering the initial library via the M13KO7 helper phage. The biopanning binding, washing, elution, and amplification procedures with SEC as the target molecule, increased the output phage titers and polyclonal phage-ELISA from the first to the third round, indicating that specific SEC-bound phage clones were significantly enriched (Figure 2A). After the third panning round via indirect phage-ELISA, 96 separate clones were chosen for phage amplification. Moreover, 47 of the 96 clones were positive, with the OD450 value of the positive colonies exceeding that of the negative control by more than three times (Figure 2(B1,B2)). BioEdit software was used to sequence and align the positive colonies, as shown in Figure 2C, where 11 positive clones exhibited high homology in the framework regions (FRs) while showing significant differences in the complementary determining regions (CDRs). The SEC-specific Nbs were categorized into 11 families according to their amino acid sequence diversity in the CDR3. The CDR3 of the 11 anti-SEC Nbs was typically 18 amino acids in length, which was considerably longer than the usual 12 amino acid CDR3 loop in the VH domains of mice. This suggests that an adequate interaction surface is created by more substantial structural flexibility, allowing the formation of various paratope structures for unique antigenic epitope recognition [29,30]. Moreover, the Nb solubility is enhanced since the Nb FRs have more hydrophilic amino acids than VH.

### 3.3. Characterization of the Binding Properties of Anti-SEC Nbs

**Figure 3:**
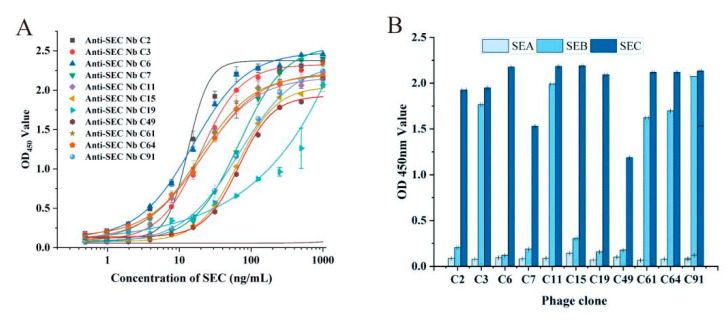
Binding activity and specific analysis of the anti-SEC Nbs. (**A**) Binding activity analysis of the phage display Nbs with SEC via phage-ELISA. (**B**) Specific analysis of the phage display Nbs with SEC via phage-ELISA.

To further evaluate the binding properties of the obtained anti-SEC Nbs with SEC, the binding activity and specificity of the 11 anti-SEC Nbs were determined with indirect ELISA. The calibration curve and limit of detection (LOD) of the indirect Nbs-ELISA were obtained as shown in Figure 3A, with LODs ranging from 0.13~11.63 ng/mL. SEA and SEB with similar structures were selected for Nb specificity analysis, while the specificity of the ELISA was evaluated according to the cross-reaction rate formula, as shown in Figure 3B. The results showed no cross-reactivity with SEA. However, a significant cross-reaction was evident with SEB and C1 (SEC1) via the antigen-binding capacity. The homology of the primary SE amino acid sequences caused cross-reactivity with monoclonal antibodies despite their serological distinction. Biological, chemical, and antigenic similarities indicate that SECs have at least 65% amino acid sequences in common with SEB while displaying a shared identity exceeding 95% [31,32,33].

### 3.4. Pairwise Interaction Analysis

The sandwich ELISA was used for pairwise coupling among the 11 Nbs, while they were also displayed by phage. The selected SEC spike concentration was 500 ng/mL, with the P/N as a parameter. According to Table 1, using the C6 anti-SEC Nb for capturing and the C11 anti-SEC Nb for detection yielded the highest P/N value. Therefore, the C11 and C6 were combined as the optimal pair for subsequent experiments.

### 3.5. Sandwich Nbs-ELISA for SEC Detection

**Figure 4:**
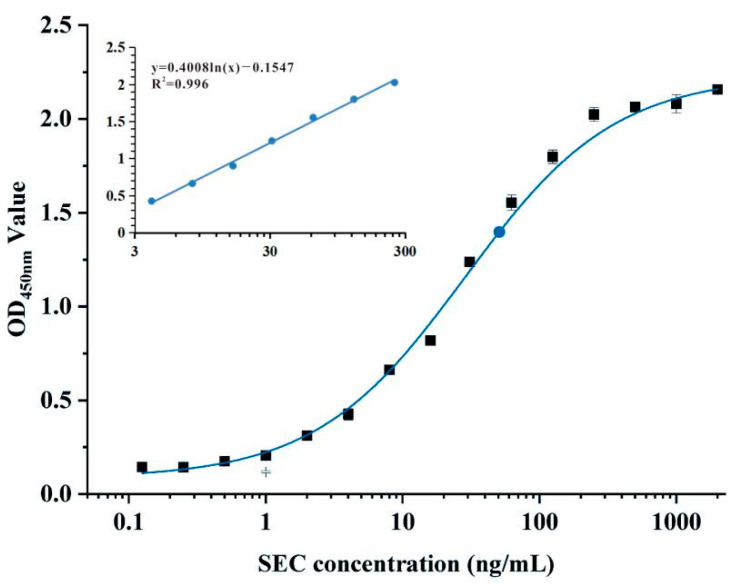
The calibration curve of the sandwich Nbs-ELISA SEC detection. Each value represents the average of three replications, as well as the standard deviations.

The sandwich Nbs-ELISA used for SEC was created using the C6 anti-SEC as the antibody for capture, and the C11 anti-SEC display phage as the antibody for detection. In optimal conditions, the C6 and C11 phage display anti-SEC Nbs were selected at 2.0 μg/mL and 1.25 × 10^8^ pfu/mL optimal concentrations, using the chessboard titration method. The calibration curve of the sandwich Nbs-ELISA was established by adding different concentrations of SEC standard solutions. The results are shown in Figure 4. The quantitative linear range was 4~250 ng/mL, exhibiting a reliable correlation coefficient (R^2^ = 0.996), while the LOD value was 2.47 ng/mL with the equation LOD = 3σ/S (where σ represents the standard deviation of the blank sample response and S denotes the calibration curve slope). Until now, minimal studies have been conducted regarding SEC detection methods. Luo developed a chemiluminescent imaging (CL) assay based on two monoclonal antibodies for the SEC1, the increased CL intensity was proportional with the concentration of SEC1 in the range of 8.0–125.0 ng/mL and the detection limit was 0.5 ng/mL. In contrast, at least 8 ng/mL of SEC1 was required to produce an unambiguous signal in ELISA (non-chemiluminescent assay) [34,35]. Wang reported a multiplexed immunochromatographic strip for simultaneous detection of SEA, SEB, SEC, SED and SEE with at concentrations as low as 2.5, 2.5, 2.5, 1, and 5 ng/mL, respectively [36]. The detection level of SEC by existing methods remains competitive compared with the results described in the literature.

### 3.6. Cross-Reactivity Assay

**Figure 5:**
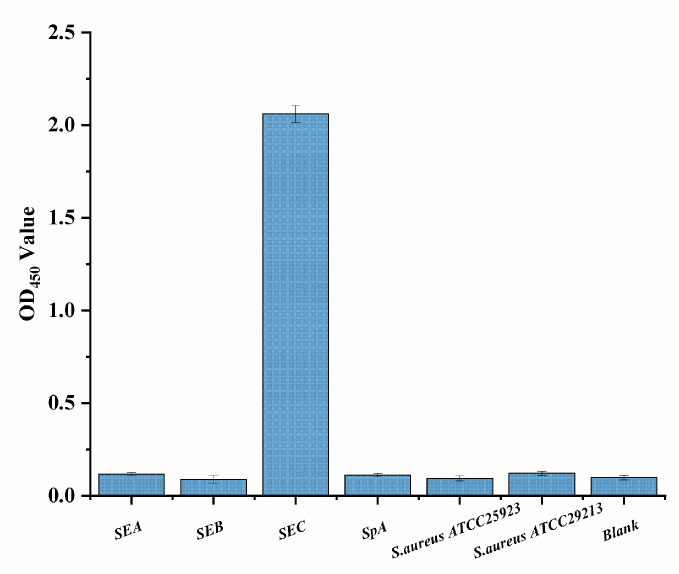
The specificity of the developed sandwich Nbs-ELISA for SEC detection. SEA, SEB, and SpA all displayed concentrations of 500 ng/mL. The *S. aureus* samples were used at equal concentrations of 10^7^ CFU/mL, while PBS buffer was used as a blank control. The error bar was the standard deviation (SD) of the samples (*n* = 3).

SEC, SEB, and SEA represent a family of structurally similar proteins produced by *S. aureus* and are the most commonly occurring enterotoxins. To ensure the specificity of the sandwich Nbs-ELISA, the cross-reaction rates were analyzed by detecting SEA (500 ng/mL), SEB (500 ng/mL), SpA (500 ng/mL), and two *S. aureus* (10^7^ CFU/mL) strains. According to Figure 5, barely any signal changes were evident in the blank sample and various other targets, demonstrating that the proposed technique displayed minimal cross-reaction with other targets.

### 3.7. Spiked Sample Analysis

Dairy products such as yogurt, cheese, and milk are frequently associated with SFP. To evaluate its application potential in dairy products, the developed ELISA was used for SEC detection in fresh milk, yogurt, and cheese purchased from a local supermarket (Yangling, China). Recovery experiments were performed via standard addition methods. The standard curve of the SEC quantitative detection showed that the dairy samples were spiked with various SEC quantities at respective 1000 ng/mL, 500 ng/mL, and 250 ng/mL concentrations. As shown in Table 2, the average SEC recovery rates ranged between 84.52% and 108.6%, with a coefficient of variation (CV) between 2.52% and 11.94%. These values satisfied the acceptance requirements (80~120%) regarding method repeatability, indicating good accuracy. Therefore, these results indicated that the sandwich Nbs-ELISA was stable and highly accurate.

## 4. Conclusions

This study develops a sensitive sandwich Nbs-ELISA to detect SEC in dairy products without the influence of SpA. This method exhibits a broad quantitative range between 4 ng/mL and 250 ng/mL with a 2.47 ng/mL LOD. The spike-and-recovery test results indicate that sandwich Nbs-ELISA is suitable for detecting SEC in dairy products. As single-domain antibodies, Nbs are highly stable, exceptionally sensitive, easily expressed, and inexpensive. However, although they naturally lack the Fc terminal of conventional monoclonal antibodies, this is not expected to restrict the immunoassay accuracy. As far as is known, no studies are available involving the use of sandwich Nbs-ELISA to detect SEC. The sandwich Nbs-ELISA shows substantial promise for detecting all SE subtypes in food samples.

## Figures and Tables

**Table 1:** Pairwise Study of Nanobodies against SEC.

Phage-Displayed Nbs	Capture Nbs (P_OD450_/N_OD450_)										
	C2	C3	C6	C7	C11	C15	C19	C49	C61	C64	C91
C2	1.14	2.03	1.52	1.12	2.08	1.08	1.81	2.07	1.29	1.06	0.81
C3	1.80	1.75	3.29	1.64	1.95	1.59	3.44	2.08	2.27	1.70	0.86
C6	1.00	1.83	1.21	0.95	1.87	0.92	1.82	1.98	1.21	1.24	0.78
C7	1.05	4.12	1.87	1.71	3.34	1.32	1.58	3.68	1.54	1.77	1.16
C11	2.74	2.32	8.08	3.53	2.15	3.34	5.61	2.42	4.00	4.43	0.94
C15	1.43	4.25	2.78	1.31	4.33	1.21	2.51	4.37	1.44	1.52	0.88
C19	0.96	3.24	1.43	0.96	2.98	1.26	2.45	2.75	1.84	1.15	0.84
C49	1.57	6.07	4.09	1.94	5.13	1.10	2.64	4.35	2.02	2.81	1.80
C61	1.71	2.45	5.57	3.84	2.61	3.25	3.35	2.43	3.00	5.22	0.90
C64	1.05	2.36	1.86	1.37	2.09	1.08	1.41	2.18	1.30	1.58	0.94
C91	1.72	7.51	5.30	2.89	6.48	1.95	3.19	7.23	2.49	3.03	0.81

**Table 2:** SEC recovery in spiked dairy products via sandwich Nbs-ELISA.

Sample	Spiked Levels (ng/mL)	Detection Level (ng/mL) (ng/mL) (*n* = 3 ^a^)	Recovery (%)
Fresh milk	250	255.22 ± 13.76	102.09 ± 5.51
	500	497.42 ± 42.47	99.48 ± 8.49
	1000	1000.05 ± 90.34	100.05 ± 9.03
Yogurt in shelf-life	250	233.79 ± 21.88	93.52 ± 8.75
	500	540.32 ± 36.27	108.06 ± 7.25
	1000	950.42 ± 119.42	95.04 ± 11.94
Cheese	250	222.81 ± 9.92	89.12 ± 3.97
	500	422.62 ± 12.62	84.52 ± 2.52
	1000	1020.48 ± 90.03	102.05 ± 9.00

^a^ Each assay was performed thrice on the same day, and each reading was taken by the same person.

## Data Availability

All the data are reported in the article.
